# Sublethal Effects of Neonicotinoid Insecticides on the Development, Body Weight and Economic Characteristics of Silkworm

**DOI:** 10.3390/toxics11050402

**Published:** 2023-04-24

**Authors:** Qiqi Chen, Shoumin Sun, Xiu Yang, Haohao Yan, Kaiyun Wang, Xiucheng Ba, Hongyan Wang

**Affiliations:** 1 Department of Plant Protection, Shandong Agricultural University, Tai’an 271018, China; qiqichen19970905@163.com (Q.C.); ssm1638946412@163.com (S.S.); yx17861507996@163.com (X.Y.);; 2 Agricultural Technology Extension Center of Binzhou, Binzhou 256600, China

**Keywords:** silkworm, imidacloprid, thiamethoxam, sublethal effects, chronic toxicity

## Abstract

Silkworm *Bombyx mori* (L.) (Lepidoptera: Bombycidae) is a critical insect for silk producers, but the inappropriate application of insecticides negatively affects the physiology and behavior of silkworms. This study found that the effects of neonicotinoid insecticides applied using two spraying methods on the growth and development of silkworms were different: the median lethal concentration (LC_50_) values of two pesticides applied using the leaf-dipping method were 0.33 and 0.83 mg L^−1^ and those of two pesticides applied using the quantitative spraying method were 0.91 and 1.23 mg kg^−1^. The concentration of pesticides on the mulberry leaves did not decrease after their application using the quantitative spraying method, and a uniform spraying density was observed after the mulberry leaves were air-dried (no liquid) under realistic conditions. We then treated silkworms with the quantitative spraying method and leaf-dipping method. The treatment of silkworm larvae with imidacloprid and thiamethoxam at sublethal concentrations significantly prolonged the development time and significantly decreased the weight and pupation rate, as well as economic indicators of enamel layers and sputum production. Thiamethoxam treatment significantly increased the activities of carboxylesterase (CarE) and glutathione-S-transferase (GST). The activity of CarE and GST increased, decreased, and then increased, and the highest activity was detected on the 10th and 12th days. Thiamethoxam exposure significantly elevated the transcription levels of *CarE-11*, *GSTe3* and *GSTz2* and induced DNA damage in hemocytes. This study confirmed that the quantitative spray method is more stable than the leaf-dipping method. Moreover, imidacloprid and thiamethoxam treatment affected the economy and indexes of silkworms and induced changes in detoxification enzymes and DNA damage in silkworms. These results provide a basis for understanding the mechanism of the sublethal effects of insecticides on silkworms.

## 1. Introduction

Silkworm (*Bombyx mori* L.), which belongs to Lepidoptera: Bombycidae, represents an economically important insect [1]. It is an important contributor to China’s economy and has been historically important for over five thousand years [2]. Currently, China has the largest area of mulberry fields. In 2017, the area of mulberry fields throughout the country was 788,733 hectares, and China produced 643,000 tons of silkworm cocoons [3]. In China, because mulberry trees, vegetables and other crops are planted in a mixed manner, mulberry leaves tend to be contaminated by insecticides, resulting in huge losses in the sericulture industry [4].

The toxicity of some pesticides to organisms, including nontarget microorganisms, has been a significant concern in recent years [5,6,7,8]. Silkworms are sensitive to adverse conditions, including pesticides, because they are maintained under isolated conditions for an extended period [9]. *B. mori* and *Plutella xylostella* (L.) (Lepidoptera: Plutellidae) have developed resistance to insecticides [10]. When exposed to low doses of pesticides, insects may develop resistance through physiological adaptation, a low cocooning rate and failure to construct a cocoon [11]. Therefore, safety evaluations of chronic pesticide exposure must be performed using silkworms. Glutathione S-transferase (GST) is a multifunctional super enzyme that plays a major role in detoxifying endogenous and exogenous compounds [12]. Some GSTs also have peroxidase and isomerase activities that react directly with reactive oxygen species, such as superoxide anions, hydrogen peroxide, and hydroxyl radicals, which induce their conversion into reduced forms and protect cells from oxidative damage [13].

Neonicotinoid insecticides are used to control sucking pests, such as cotton aphids, on a variety of crops [14,15]. These pesticides act on the nervous system of insects by binding to nicotinic acetylcholine receptors (nAChRs), which results in sustained stimulation that leads to paralysis or death [16]. During the field application of thiamethoxam, the drug often drifts and causes harm to nontarget organisms, which leads to a variety of problems. As a result, the use of thiamethoxam is being gradually controlled [17]. Studies have found that thiamethoxam has an isolation effect on F1 individuals of cotton aphids due to changes in growth and development and the intermittent expression of detoxification-related genes [18]. The main varieties of neonicotinoid insecticides include thiamethoxam, dinotefuran, imidacloprid, thiacloprid, acetamiprid, clothianidin, and nitenpyram [16]. The toxicity of neonicotinoids has been studied by numerous researchers. Studies have shown that the toxicity of acetamidine to silkworms is complex and serious, which can lead to disturbance of the reproductive system in silkworms, affect the reproduction of the next generation and produce a variety of toxic reactions, including those catalyzed by detoxification enzymes [19,20]. Previous studies on the acute toxicity of imidacloprid have found that it is highly toxic to silkworms [21]. Exposure to dinotefuran has been found to damage the cells of silkworms and partially inhibit many metabolic processes in the body, which affects the normal growth and development of silkworms [22]. Moreover, acetamiprid potentially impacts caterpillars of *B. mori* [20].

This study was carried out following standard practices based on the following objectives: (1) to assess the chronic effects of thiamethoxam and imidacloprid applied to silkworms using two methods; (2) to assess the effects of thiamethoxam on the enzyme activities of carboxylesterase (CarE) and glutathione-S-transferase (GST); (3) to assess the effects of thiamethoxam on hemocyte DNA damage.

## 2. Materials and Methods

### 2.1. Insect strain

Among Chinese silkworm farming populations, the Chun Lei × Zhen Zhu strain is susceptible to many pesticides and was acquired from Shandong Guangtong Silkworm Eggs Co. Ltd. (Qingzhou, Shandong Province, China) [23]. The silkworms were obtained as eggs and stored in a standard refrigerator. After egg hatching, the laboratory conditions were set as follows: temperature of 25–26 °C, 70–85% relative humidity (RH), and 16 h light: 8 h dark cycle [24,25,26]. The larvae were fed 4 times per day with clean mulberry of the same quality and not exposed to pesticides. Second-instar larvae with the same size and weight were collected for laboratory tests.

### 2.2. Chemicals

The chemicals used in the experiment were imidacloprid (CAS: 105827-78-9; purity: 94.8%) and thiamethoxam (CAS: 153719-23-4; purity: 95%), which were provided by Shandong Zhongnong United Biotechnology Co., Ltd. (Ji’nan, Shandong, China). Stock solutions (0.1%) of imidacloprid and thiamethoxam were prepared with ddH_2_O.

### 2.3. Toxicity Bioassay

Clean mulberry leaves were immersed in specific pesticide solutions for 10 s [27]. The air-dried and treated mulberry leaves were laid flat in glass culture dishes. Collected larvae were carefully placed on the upper surfaces of the leaves and allowed to feed on the treated mulberry leaves for 96 h. The mortality was recorded after 96 h of exposure. In addition, fresh mulberry leaves (2 to 3 g) were sprayed with imidacloprid and thiamethoxam. The pesticide solutions (0.125 mg/L, 0.25 mg/L, 0.50 mg/L, 1 mg/L and 2 mg/L) were evenly sprayed onto the lower surfaces of the leaves. The mulberry leaves were individually weighed, and the exact quantity of pesticide applied to each leaf was determined. The treated leaves were placed flat in culture dishes with the treated facing up. Three replicates were included in the experiment, and each replicate contained twenty silkworm larvae. Twenty silkworm larvae were carefully placed on top of each leaf using a soft brush. Glass culture dishes with a diameter of 15 cm and a height of 3 cm were used as exposure containers. The culture dishes were placed in a climate-controlled incubator with the same rearing conditions as those mentioned above. The mortality was recorded after 96 h of exposure.

### 2.4. Chronic Toxicity Test

The two pesticides were to obtain 1 g/L stock liquid solutions, and gradient dilution was carried out with ddH_2_O. Based on the LC_50_ values of the pesticides determined in this study, five pesticide concentrations with acute toxicity LC_50_ of values 1/400, 1/200, 1/100, 1/50 and 1/20 were established. The experiment was completely randomized; 3 biological replicates of each experimental group were included, and each experimental group contained 10 silkworms. The symptoms of silkworm poisoning and complications after poisoning were observed. Five concentrations were established in the preliminary experiment, and blanks were included. The solubilizing agent ddH_2_O was added when necessary to help dissolve the solution. Each treatment or control experiment was performed three times, and ten domestic silkworms were included in each treatment group.

Quantitative spraying method: Fresh mulberry leaves with luster were selected from the top of the tree. Two to three grams of young leaves were weighed, and two milliliters of different concentrations of pesticides were sprayed onto the backs of the leaves with a laboratory spray tower. The leaves were air-dried for 5~10 min. Each primary leaf vein was inserted into a centrifuge tube (1.5 mL) with agar for moisture preservation, and the leaves were placed into culture dishes. Second-instar silkworm larvae were transferred to dishes with mulberry leaves and allowed to feed on the mulberry leaves until the end of the second-instar larval stage. The larvae were fed on nontoxic mulberry leaves from the third-instar larval stage to maturity.

Leaf-dipping method: Fresh mulberry leaves with luster were selected from the top of the tree. Two to three grams of young leaves were weighed, and the leaves were sprayed with water. The surface water was air-dried, and the leaves were dipped in liquid pesticide for 10 s. After natural drying, the leaves were placed into culture dishes. Second-instar silkworm larvae were randomly transferred into the culture dishes containing mulberry leaves and allowed to feed until the end of the second-instar larval stage. The larvae were fed on nontoxic mulberry leaves from the third-instar larval stage to maturity.

### 2.5. Enzyme Activity Determination

Mulberry leaves were treated with chronic test concentrations of thiamethoxam using the spraying method. Silkworms from the 2nd- to 3rd-instar stage were continuously fed treated mulberry leaves, or untreated mulberry leaves were used. During the experiment, samples were collected every other day, and the activity of CarE and GST in the silkworms was measured.

In the assessment of CarE activity, α-naphthyl acetate was used as the substrate. α-Naphthol (μmol) was used as the abscissa, and the OD value was used as the ordinate to draw the standard curve. The samples to be tested were thoroughly ground in liquid nitrogen and transferred to a centrifuge tube, and 5 mL of 0.04 mol/L phosphate buffer (pH 7.0) was added. The samples were then centrifuged at 8000 r/min for 10 min in a high-speed refrigerated centrifuge. The centrifuge tube was removed, and the supernatant was used as the enzyme source for the measurement of enzyme activity. Five milliliters of 3 × 10^−4^ mmol/L substrate was added to the test tube, and the tube was then incubated at 25 °C for 5 min. Then, 1 mL of enzyme solution was added, and 1 mL of 0.04 mol/L pH 7.0 phosphate buffer was added to the control. Each test tube was shaken in a water bath at 30 °C for 30 min, and 1 mL of color reagent was added to stop the reaction. After mixing, the solution in the test tube was allowed to stand at room temperature for 30 min. Three replicates were included in the experiment.

The amount of α-naphthol produced in the reaction was calculated according to the standard curve, divided by the reaction time (30 min), and then divided by the volume of enzyme solution (1 mL in this experiment) to obtain the amount of hydrolyzed α-naphthyl acetate per milliliter of enzyme solution per minute (μmol/min/mL).

For the assessment of GST activity, 2,4-dinitrochlorobenzene was used as the substrate. The samples to be tested were thoroughly ground in liquid nitrogen and transferred to a centrifuge tube, and 1 mL of phosphate buffer (0.066 mol/L, pH = 7.0) was added. The solution was then centrifuged in a high-speed refrigerated centrifuge at 8000 r/min for 10 min. The centrifuge tube was removed, and the supernatant was used as the enzyme source for the measurement of enzyme activity. The following reagents were sequentially added to the test tube: 2.4 mL of 0.066 mol/L phosphate buffer (pH 7.0), 0.3 mL of 0.05 mol/L reduced glutathione solution, 0.1 mL of 0.03 mol/L CDNB and 0.2 mL of enzyme solution (0.2 mL of buffer was used instead of enzyme solution for the control). After the reagents were mixed, each test tube was placed in a water bath at 27 °C for 5 min. The OD value was recorded every 30 s under an ultraviolet spectrophotometer at 340 nm, and the change in the OD value over 5 min was recorded. Each treatment was repeated 3 times.

The activity of GST was calculated with the following formula: GST activity unit (nmol/min) = (ΔOD340·v)/(ε·L), where ΔOD340 is the change in light absorption per minute (ΔOD340/min), v is the enzymatic reaction volume, ε is the extinction coefficient of the product, and L is the optical path of the solution. The specific activity of GST (mmol/min/mg Pr.) is the ratio of the activity of GST to the protein content of the enzyme solution.

### 2.6. RNA Extraction and Quantitative Real-Time PCR (qRT–PCR)

Silkworms exposed to 1/100 LC_50_ of thiamethoxam for 96 h were collected and stored at −80 °C. Total RNA was extracted using an RNA extraction reagent kit (Vazyme Biotech Co., Ltd., Nanjing, China). The RNA quantity was determined spectrophotometrically, and its quality was assessed by formaldehyde agarose gel electrophoresis. cDNA was synthesized using a reverse transcriptase kit (Vazyme Biotech Co., Ltd., Nanjing, China). A standard curve was drawn using a series of standards with known initial copy numbers to establish a linear relationship between the Ct value and the logarithm of the initial template amount. The detailed detection procedures were as follows: predenaturation at 95 °C for 3 min and 40 cycles of denaturation at 95 °C for 10 sand annealing/extension at 60 °C for 30 s. The relative expression analysis was performed in triplicate. Specific primers were designed using Primer Express software (Sandon Biotech, Shanghai, China) (Table 1).

### 2.7. Hemocytes DNA Damage

Leaves with fifth-instar silkworm leaves were treated with thiamethoxam at 1/400 LC_50_ and 1/100 LC_50_. After 5 days, we prepared glass slides using cell extract, low melting point agarose, and high melting point agarose. The silkworm horn was cut, and hemolymph was collected. PBS buffer (the main components of Na_2_HPO_4_, KH_2_PO_4_, NaCl and KCl) (2000 r/min, 4 °C, 20 min) was then added, the lymphocyte layer was extracted, and physiological saline was added to prepare a hemolymph cell suspension; the suspension was stored at 4 °C. The DNA damage experiment was performed using a previously described method [28].

At 60 °C, 150 μL of 0.8% normal melting point agarose was set, a clean cover glass was added, the glass was cooled with an ice pack, and the cover glass was slowly removed. At 37 °C, 10 μL of the cell suspension was mixed with 100 μL of 1.0% low melting point agarose; the mixture was rapidly dropped onto the first layer of agarose, a cover glass was added to evenly distribute the mixed liquid, and the mixture was maintained at 4 °C. After the second layer of agarose solidified, the cover glass was removed, 50 μL of 0.5% low melting point agarose was added dropwise, a cover glass was rapidly placed, and the cover glass was removed after solidification at 4 °C. The slides were immersed in lysis solution at 4 °C for 1 h and rinsed with clean water. The glass slide was placed horizontally on the anode of the electrophoresis tank. The electrophoresis tank contained 400 mL of precooled alkaline electrophoresis buffer (300 mmol/L NaOH, 1.0 mmol/L), which covered the glass slide with 2.5 mm of a buffer. The slide was placed in the buffer for 30 min. The settings for the electrophoresis instrument were as follows: 25 V and 300 mA for 15 min. After electrophoresis, the slides were rinsed 3 times with distilled water, and filter paper was used to absorb excess water. The slides were then placed in a small porcelain dish, and 0.4 mol/L Tris-HCl buffer solution (pH = 7.5, 4 °C) was added. The slides were immersed in neutralizing reaction buffer for 15 min. After the removal of Tris-HCl, absolute ethanol was slowly added, and the slides were immersed for 1 h at 4 °C for dehydration. After dyeing, 30 μL of EB water solution was added for 15 min, the slides were rinsed twice with deionized water, and excess water was removed. The glass slide was placed under a fluorescence microscope (Shinagawa Intercity Tower C, 2-15-3, Konan, Minato-ku, Tokyo, 108-6290, Japan) with a wavelength of 515–560 nm. The extent of DNA damage was quantified based on the olive tail moment (OTM) [29].

### 2.8. Statistical Analyses

We used SPSS 18.0 software to perform a multifactor analysis of variance, and the least significant difference (LSD) method was applied after the preliminary analysis. Probit analysis was used to calculate the LC_50_ values. The data were subjected to Shapiro–Wilk’s and Levene’s tests (*p* > 0.05) to confirm whether the data adhered to a normal distribution and homoscedasticity. To reduce errors during the test, the whole cocoon weights and cocoon layer weights for each group and the blank control were averaged and considered an internal factor. These data were then compared with the internal coefficient ratio as the dependent variable.

## 3. Results

### 3.1. Bioassay Evaluation

The similarities and differences between the two acute toxicity test methods are shown in Table 2. With the leaf-dipping method, the LC_50_ values of imidacloprid and thiamethoxam were 0.33 and 0.83 mg L^−1^, respectively, and the LC_50_ values of imidacloprid and thiamethoxam applied using the quantitative spraying method were 0.91 and 1.23 mg kg^−1^, respectively.

### 3.2. Chronic Toxic Effects on B. mori

**Figure 1:**
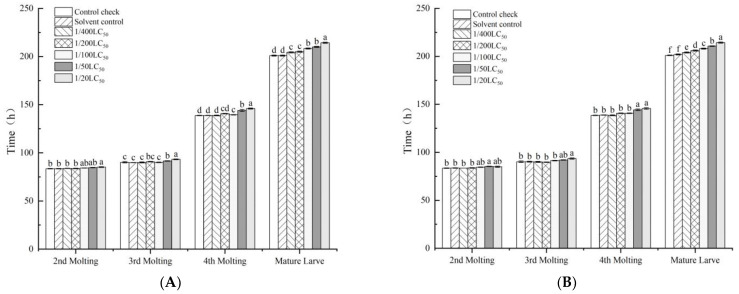
Effect of the leaf-dipping method with imidacloprid and thiamethoxam on the silkworm developmental period. (**A**) imidacloprid; (**B**) thiamethoxam. Note: Values are means (±SEM) of the time spent in each molting by the leaf-dipping method. Significant differences are represented between different letters according to the least significant difference (LSD) method (*p* < 0.05).

**Figure 2:**
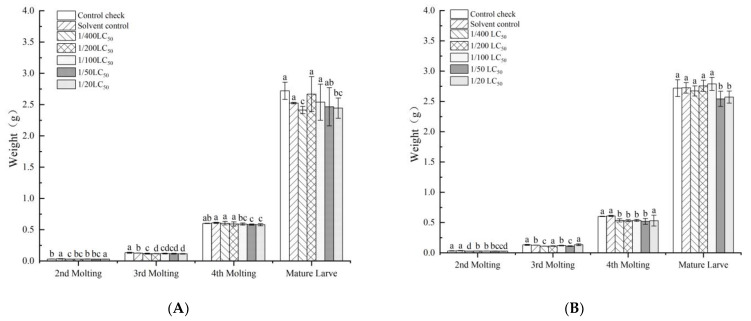
Effect of the leaf-dipping method of imidacloprid and thiamethoxam on body weight of silkworm cocoons. (**A**) imidacloprid; (**B**) thiamethoxam. Note: Values are means (±SEM) of body weight of silkworm in each molting by the leaf-dipping method. Significant differences are represented between different letters according to the least significant difference (LSD) method (*p* < 0.05).

**Figure 3:**
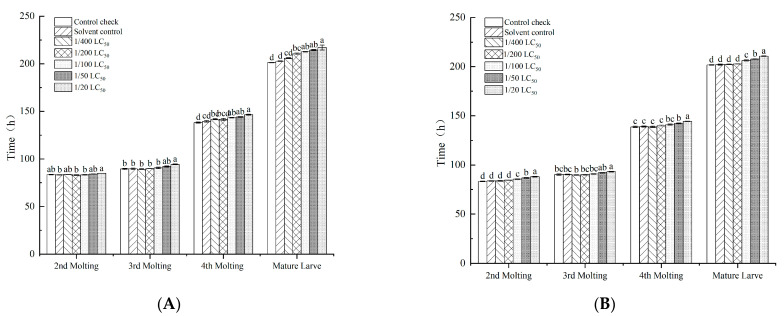
Effect of the quantitative spraying method with imidacloprid and thiamethoxam on the silkworm developmental period. (**A**) imidacloprid; (**B**) thiamethoxam. Note: Values are means (±SEM) of the time spent in each molting by the quantitative spraying method. Significant differences are represented between different letters according to the least significant difference (LSD) method (*p* < 0.05).

The leaf-dipping method affected the silkworm growth phase, and the developmental time of silkworms increased with increases in the pesticide dose (Figure 1). Similarly, the leaf-dipping method was found to have an effect on body weight, but significant differences in the silkworm between the treatment and control groups were not observed with increasing doses (Figure 2). At increasing neonicotinoid insecticide doses, significant differences were not observed in the body weight of the total sputum or the number of sputum layers. Nevertheless, the rate of sputum production decreased (Table 3). With the quantitative spraying method, the developmental time of each instar increased as the neonicotinoid insecticide dose increased (Figure 3).

**Figure 4:**
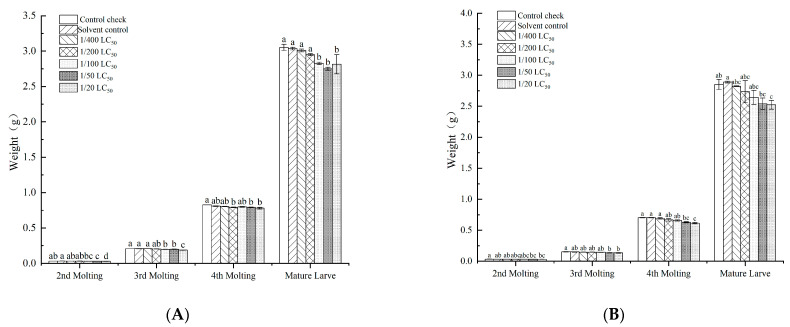
Effect of the quantitative spraying method with imidacloprid and thiamethoxam on body weight of silkworm cocoons. (**A**) imidacloprid; (**B**) thiamethoxam. Note: Values are means (±SEM) of body weight of silkworm in each molting by the quantitative spraying method. Significant differences are represented between different letters according to the least significant difference (LSD) method (*p* < 0.05).

The quantitative spraying method affected the weight of silkworms, and the silkworm weight at each instar gradually decreased as the neonicotinoid insecticide dose increased (Figure 4). Imidacloprid affected the economic indicators of silkworms treated with the quantitative spraying method. With increases in the neonicotinoid insecticide doses, the number of silkworms, enamel layers, scar rate and replacement rate gradually decreased (Table 4).

### 3.3. Effect on the Activities of Enzymes

**Figure 5:**
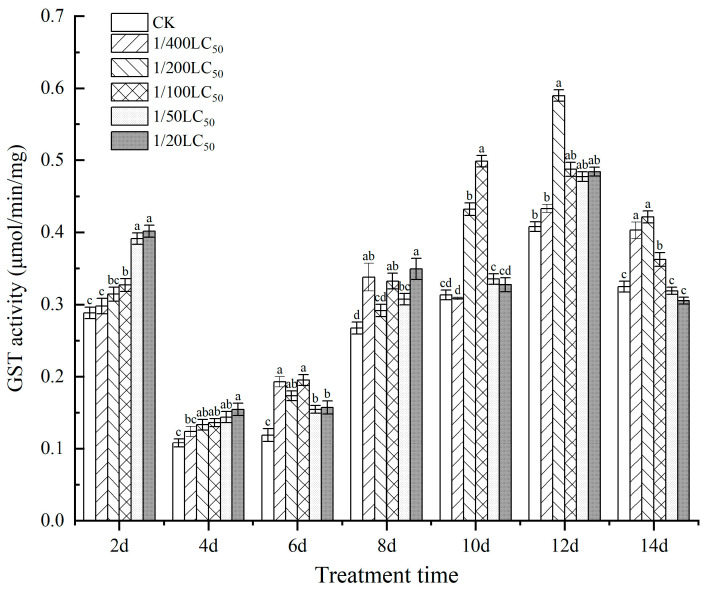
The effect on the activities of GST in silkworms after thiamethoxam treatment. Data in the same column followed by different lowercase letters in the table indicate significant differences at 0.05 levels. Different letters indicate significant differences between treatments of the same day (*p* < 0.05).

**Figure 6:**
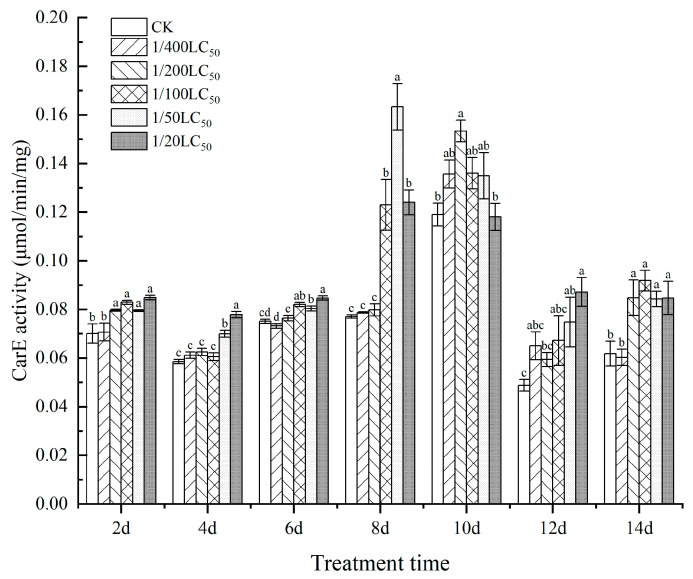
The effect on the activities of CarE in silkworms after thiamethoxam treatment. Data in the same column followed by different lowercase letters in the table indicate significant differences at 0.05 levels. Different letters indicate significant differences between treatments of the same day (*p* < 0.05).

As shown in Figure 5 and Figure 6, the CarE and GST activities in silkworms treated with thiamethoxam showed trends of first increasing, then decreasing, and finally increasing over time, and the highest activity was detected on the 10th day. On day 6, the GST activity values of each treatment group were significantly different from that of the control group. The GST activity 1/400 LC_50_ group was 1.62-fold that of the CK group, and that of the 1/100 LC_50_ group was 1.64-fold that of the CK group. On day 10, the GST activity of the 1/100 LC_50_ group was 1.59-fold that of the CK group. On day 12, the GST activity of the 1/200 LC_50_ group (the highest value detected during the CarE activity experiment) was 1.45-fold that of the CK group.

Moreover, the CarE activity of the 1/50 LC_50_ group was 2.12-fold that of the CK group on day 8. Moreover, on day 10, the CarE activity of the 1/200 LC_50_ group was 1.29-fold that of the CK group. The results indicated that low concentrations of thiamethoxam could influence CarE and GST activities.

### 3.4. Effects of Thiamethoxam on the Transcription of Detoxification Enzyme Genes

**Figure 7:**
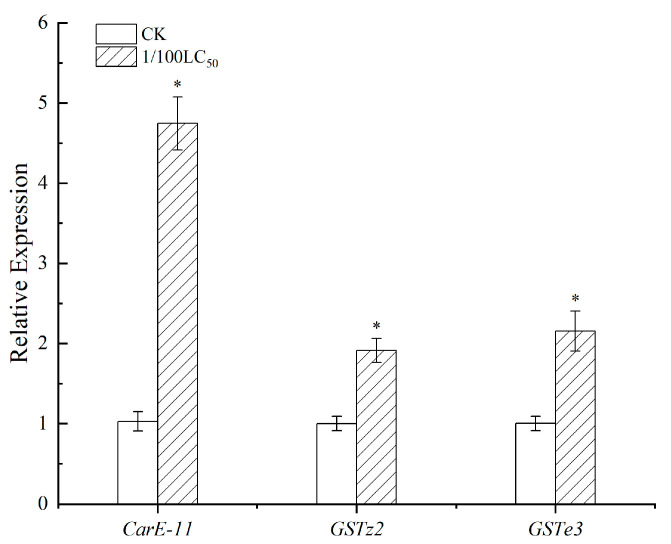
Effects of thiamethoxam on expression of detoxification genes in the midgut of silkworms. Transcription levels of carboxylesterase (CarE) family gene (CarE-11) and two glutathione S-transferase (GST) genes (GSTe3 and GSTz2). Data are shown as mean ± SEM. The significance of the difference is indicated and expressed as *. (*p* < 0.05).

To investigate the mechanisms underlying the effects of low-dose thiamethoxam, we selected three genes (*CarE-11*, *GSTe3* and *GSTz2*). The results showed that the transcription level of the CarE gene *CarE-11* after 96 h of exposure to thiamethoxam was significantly higher than that of the control group, and the relative expression of *CarE-11* at 96 h was 4.61-fold. The expression of the GST genes *GSTe3* and *GSTz2* was also elevated after exposure to this pesticide. The expression of *GSTz2* showed 1.91-fold upregulation at 96 h, and that of *GSTe3* was increased by 2.15-fold at this time point (*p* <0.05, Figure 7).

### 3.5. DNA Damage

**Figure 8:**
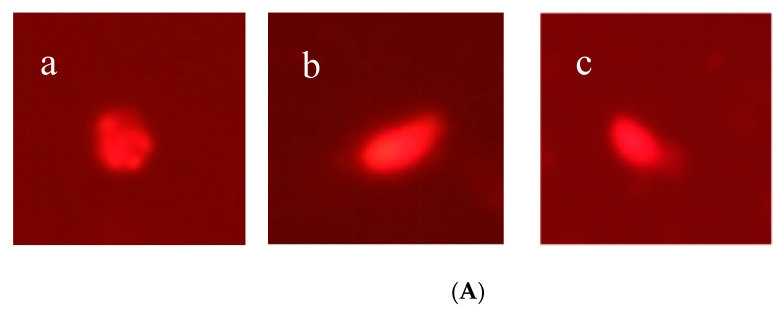

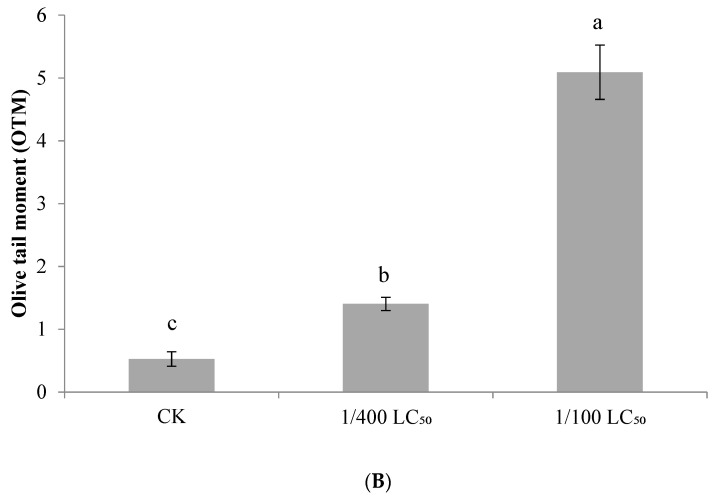
Comet experiment of thiamethoxam in silkworms. (**A**) Comet test of silkworm body cells under different concentrations of thiamethoxam, (**a**) CK, (**b**) nuclei of silkworms under 1/400 LC_50_ of thiamethoxam, (**c**) nuclei of silkworms under 1/100 LC_50_ of thiamethoxam. (**B**) The effect of exposure to different concentrations of thiamethoxam on the comet olive tail moment (OTM) of the silkworm body cavity cells in 5 days, the significant differences are represented between different letters (*p* < 0.05).

Even at low concentrations, thiamethoxam can cause DNA damage (Figure 8). The control group showed round and bright spots, but both groups treated with two concentrations of thiamethoxam showed tails. The OTM value increased with increases in the concentrations of thiamethoxam. On day 5, the OTM value of the silkworm coelomocyte comets treated with 1/400 LC_50_ of thiamethoxam was 1.3262, which was 18.68-fold that of the CK group. The OTM of the silkworm coelomocyte comets treated with 1/100 LC_50_ of thiamethoxam was 4.0810, which was 3.07-fold that obtained with 1/400 LC_50_ of thiamethoxam. The results indicated that a low concentration of thiamethoxam could cause damage to DNA in silkworms.

## 4. Discussion

*B. mori* is an important economic pest of mulberry in the field and is also greatly affected by pesticide exposure [26]. Imidacloprid and thiamethoxam are widely used to control aphids, rice plant hoppers and other pests. The effective dosages of imidacloprid and thiamethoxam for the control of rice plant hoppers are usually 46.7 to 93.3 mg/L and 16.7 to 33.3 mg/L, respectively [30]. Compared with our acute toxicity test results, the dosages of the two pesticides were both of high risk for silkworms.

This research aimed to study the chronic effects of thiamethoxam and imidacloprid applied using the leaf-dipping and quantitative spraying methods on silkworms and to compare the advantages and disadvantages of these two methods based on chronic toxicity tests in silkworms. The effective dosages of imidacloprid and thiamethoxam for the control of rice plant hoppers are usually 46.7 to 93.3 mg/L and 16.7 to 33.3 mg/L, respectively [31]. A previous study found that an appropriate concentration of imidacloprid had a certain positive effect on silkworms and that a concentration of this pesticide less than or higher than this amount inhibits silkworms [29]. The fertility of *Sitobion avenae* feeding on wheat plants treated with LC_10_ (0.0053 mg/L) of imidacloprid, dinotefuran, thiacloprid and thiamethoxam is not affected [32]. Studies have found that thiamethoxam at a sublethal concentration has no obvious stimulating effect on the adult longevity of aphids and can be used as a permitted level for crop application [33]. However, our study showed that imidacloprid had a negative effect on silkworms regardless of the applied amount and that a higher concentration had a greater negative impact. Exposure to thiamethoxam also had adverse effects on the growth and development, enzyme activity and DNA of silkworms. Therefore, the findings recommend avoiding the use of these two insecticides during the periods of mulberry picking and silkworm rearing. The LC_50_ values of the two pesticides applied using the leaf-dipping method were 0.33 and 0.83 mg liter^−1^, respectively, whereas the LC_50_ values of these two pesticides applied using the quantitative spraying method were 0.91 and 1.23 mg kg^−1^, respectively. The results also showed that the concentrations of pesticides had significant effects on various developmental indexes, indicating that the method of pesticide application had certain effects on the developmental indexes of silkworms. The pesticide concentrations and application methods significantly affected developmental indicators.

Acute and chronic toxicity experiments are the standard methods for evaluating the effect of pesticides on silkworms. In the chronic toxicity test, the effect of leaf dipping on silkworm growth was greater than that of quantitative spraying. Arakawa et al. [34] showed that a small amount of iminoctadine prolonged the developmental phase of silkworms and increased the weight loss of molted silkworms, and these results were also observed with the quantitative spraying method in the present study. With the leaf-dipping method, the development time of the silkworms was prolonged, and the weight of the molted silkworms decreased; however, the weight fluctuation of the molted silkworms and the decrease in economic indicators were not obvious.

GST is involved in the detoxification metabolic mechanism of thiamethoxam. CarE plays a role in insect resistance in the presence of thiamethoxam [35]. CarE and GST are regulated by the CncC/Keap1-ARE signaling pathway [36] and thus play an important role in the silkworm body. Understanding the impact of pesticides on these enzymes is beneficial to the improved use of pesticides. In addition, we assessed the expression of genes related to CarE and GST enzyme activities, including *CarE-11*, *GSTz2* and *GSTe3*. The results showed that exposure to low doses of thiamethoxam increased the expression levels of *CarE-11*, *GSTz2* and *GSTe3* and the activities of related enzymes.

In addition, we also assessed cell-DNA-damage-related indicators. DNA damage is one of the important parameters for evaluating the genotoxicity of exogenous poisons. The comet assay has been widely used to detect DNA damage in single cells and determine the genotoxicity of exogenous poisons. The comet assay demonstrated a dose–response relationship between pesticides and DNA damage [37]. Shen et al. [38] found that a low dose of abamectin causes significant DNA damage and genotoxicity to *Bombyx mori* blood cells, and the degree of damage is related to dose and time. In particular, mitochondrial DNA is more vulnerable to exogenous damage than nuclear DNA, and oxidative damage and mitochondrial damage interact to form a vicious cycle [39,40,41]. In this study, we found that thiamethoxam could damage the blood cell DNA of silkworms in a dose-dependent manner, consistent with previous research results. Studies have observed genotoxicity after exposure to thiamethoxam, even at low doses. If the activity of detoxification enzymes in the silkworm is too high and exceeds the detoxification limit, the excessive level of toxic substances in the silkworm may cause the cells to produce harmful substances and cause DNA damage. For example, DNA is easily damaged under free radical attack [31]. Once the cells are damaged, the transport, transformation and retention of various substances in the cells will be affected, which will influence the growth and development of silkworms.

## 5. Conclusions

Bioassays could be used to illustrate the chronic effects of pesticide exposure effectively. The two tested neonicotinoid insecticides exert significant chronic effects on silkworms during the growth phase. The results showed that applying pesticides using the leaf-dipping method for the assessment of chronic toxicity in silkworms is a simple but time-consuming method and presents poor precision and reproducibility. In contrast, the quantitative spraying method exhibits high precision and high precision reproducibility during realistic experiments. A low concentration of thiamethoxam was found to have the ability to affect CarE and GST activities and cause damage to DNA in silkworms. When this agent is used to control rice plant hoppers in paddy fields, it can easily drift, cause harm to silkworms and affect production. Therefore, appropriate scheduling of the application time of this pesticide is essential.

## Figures and Tables

**Table 1:** List of primers used in this study.

Genes	Primers
*Actin3*	F: 5′-CGGCTACTCGTTCACTACC-3′
	R: 5′-CCGTCGGGAAGTTCGTAAG-3′
*GSTz2*	F: 5′-TTGGCTGTCTTCGTGTTC-3′
	R: 5′-GCTGGATTGATGGCTCTG-3′
*GSTe3*	F: 5′-GAAGATGGCGACCTGATTA-3′
	R: 5′-GAGAACAACACCGTGCTA-3′
*CarE-11*	F: 5′-AATGCCAGTGCTAATAGGTT-3′
	R: 5′-CGTCTAGTTCTTCGTTCGTA-3′

**Table 2:** The 96 h LC_50_ values and 95% relative confidence intervals of imidacloprid and thiamethoxam against *B. mori*.

Pesticides	Methods	Toxicity Regression Equations	LC_50_ Values	R^2^	95% RCIs
Imidacloprid	Leaf-dipping method	Y = 1.28 + 2.82X	0.33 mg L^−1^	0.992	0.27–0.40
	Quantitative spraying method	Y = 0.11 + 2.60X	0.91 mg kg^−1^	0.998	0.63–1.34
Thiamethoxam	Leaf-dipping method	Y = 0.31 + 3.72X	0.83 mg L^−1^	0.990	0.69–1.00
	Quantitative spraying method	Y = −0.21 + 2.53X	1.23 mg kg^−1^	0.997	0.97–1.59

Note: mg L^−1^ for the leaf-dipping method and mg kg^−1^ for the quantitative spraying method. The 96 h LC_50_ values of imidacloprid and thiamethoxam on silkworms and 95% relative confidence intervals (RCIs) were calculated by using SPSS 18.0 software.

**Table 3:** Effect of the leaf-dipping method with imidacloprid and thiamethoxam on the main economic characteristics of silkworms.

Pesticides	Reagent Concentration	Cocoon Weight/g	Cocoon Shell Weight/g	Pupation Rate/%	Dead Cage Rate/%
imidacloprid	Control check	1.401 ± 0.057 a	0.281 ± 0.009 bc	100.000 a	0 c
	Solvent control	1.339 ± 0.016 cd	0.271 ± 0.009 cd	100.000 a	0 c
	1/400 LC_50_	1.359 ± 0.021 bc	0.267 ± 0.002 d	100.000 a	0 c
	1/200 LC_50_	1.365 ± 0.025 bc	0.290 ± 0.017 a	100.000 a	0 c
	1/100 LC_50_	1.324 ± 0.121 bc	0.264 ± 0.033 d	81.670 ± 1.667 b	18.400 bc
	1/50 LC_50_	1.288 ± 0.133 d	0.270 ± 0.027 d	78.330 ± 1.667 c	21.700 a
	1/20 LC_50_	1.383 ± 0.014 ab	0.290 ± 0.006 ab	76.670 ± 1.667 c	23.300 a
thiamethoxam	Control check	2.720 ± 0.139 bc	0.281 ± 0.009 b	100.000 a	0 e
	Solvent control	2.724 ± 0.087 b	0.288 ± 0.009 ab	100.000 a	0 e
	1/400 LC_50_	2.673 ± 0.084 bc	0.283 ± 0.019 b	100.000 a	0 e
	1/200 LC_50_	2.758 ± 0.092 b	0.289 ± 0.012 ab	90.000 ± 1.667 b	10.000 d
	1/100 LC_50_	2.789 ± 0.108 a	0.293 ± 0.002 a	81.670 ± 1.667 c	19.600 c
	1/50 LC_50_	2.542 ± 0.126 c	0.264 ± 0.018 c	76.670 ± 1.667 d	23.400 b
	1/20 LC_50_	2.571 ± 0.099 a	0.272 ± 0.014 b	73.330 ± 1.667 e	26.700 a

Note: Record the main economic characteristics (cocoon weight and cocoon shell weight) of silkworms at different reagent concentrations by the leaf-dipping method. Values are means (±SEM) of the experiments. Significant differences are represented between different letters according to the least significant difference (LSD) method (*p* < 0.05).

**Table 4:** Quantitative effects of the quantitative spraying method with imidacloprid and thiamethoxam on the main economic characteristics of silkworms.

Pesticides	Reagent Concentration	Cocoon Weight/g	Cocoon Shell Weight/g	Pupation Rate/%	Dead Cage Rate/%
imidacloprid	Control check	1.784 ± 0.008 a	0.319 ± 0.001 a	100.000 ± 0 a	0 b
	Solvent control	1.784 ± 0.089 a	0.316 ± 0.003 a	100.000 ± 0 a	0 b
	1/400 LC_50_	1.772 ± 0.014 a	0.310 ± 0.003 a	100.000 ± 0 a	0 b
	1/200 LC_50_	1.783 ± 0.015 a	0.312 ± 0.004 a	100.000 ± 0 a	0 b
	1/100 LC_50_	1.761 ± 0.012 a	0.306 ± 0.002 a	100.000 ± 0 a	6.700 ab
	1/50 LC_50_	1.606 ± 0.039 b	0.280 ± 0.006 b	90.000 ± 0 a	9.900 ab
	1/20 LC_50_	1.519 ± 0.048 b	0.265 ± 0.004 b	86.700 ± 1.667 b	15.700 a
thiamethoxam	Control check	1.383 ± 0.008 ab	0.288 ± 0.003 a	100.000 ± 0 a	0 c
	Solvent control	1.403 ± 0.039 a	0.283 ± 0.001 a	100.000 ± 0 a	0 c
	1/400 LC_50_	1.357 ± 0.006 ab	0.287 ± 0.002 a	100.000 ± 0 a	0 c
	1/200 LC_50_	1.342 ± 0.026 ab	0.280 ± 0.004 a	100.000 ± 0 a	0 c
	1/100 LC_50_	1.292 ± 0.020 ab	0.251 ± 0.001 b	91.670 ± 1.667 b	7.600 bc
	1/50 LC_50_	1.287 ± 0.011 ab	0.257 ± 0.004 b	86.670 ± 1.667 c	11.400 ab
	1/20 LC_50_	1.271 ± 0.021 b	0.252 ± 0.005 b	83.330 ± 1.667 d	16.800 a

Note: Record the main economic characteristics (cocoon weight and cocoon shell weight) of silkworms at different reagent concentrations by the quantitative spraying method. Values are means (±SEM) of the experiments. Significant differences are represented between different letters according to the least significant difference (LSD) method (*p* < 0.05).

## Data Availability

Data are available from the corresponding author upon reasonable request.
